# Influence of Aluminum and Copper on Mechanical Properties of Biocompatible Ti-Mo Alloys: A Simulation-Based Investigation

**DOI:** 10.3390/mi14051081

**Published:** 2023-05-20

**Authors:** Omid Ashkani, Mohammad Reza Tavighi, Mojtaba Karamimoghadam, Mahmoud Moradi, Mahdi Bodaghi, Mohammad Rezayat

**Affiliations:** 1 Department of Materials Science and Engineering, Faculty of Engineering, Islamic Azad University, Science and Research Branch, Tehran 1477893855, Iran; 2 Department of Materials Science and Engineering, Faculty of Engineering, Islamic Azad University, Karaj 3149968111, Iran; taavighi.mreza@gmail.com; 3 Department of Mechanics, Mathematics and Management, Polytechnic University of Bari, Via Orabona 4, 70125 Bari, Italy; 4 Faculty of Arts, Science and Technology, University of Northampton, Northampton NN1 5PH, UK; mahmoud.moradi@northampton.ac.uk; 5 Department of Engineering, School of Science and Technology, Nottingham Trent University, Nottingham NG11 8NS, UK; 6 Center for Structural Integrity, Micromechanics, and Reliability of Materials (CIEFMA)-Department of Materials Science and Engineering, Universitat Politècnica de Catalunya-BarcelonaTECH, 08019 Barcelona, Spain

**Keywords:** Ti–xCu–xMo, Ti-9Mo, antibacterial titanium alloy, simulation, metallic biomaterial

## Abstract

The use of titanium and titanium-based alloys in the human body due to their resistance to corrosion, implant ology and dentistry has led to significant progress in promoting new technologies. Regarding their excellent mechanical, physical and biological performance, new titanium alloys with non-toxic elements and long-term performance in the human body are described today. The main compositions of Ti-based alloys and properties comparable to existing classical alloys (C.P. TI, Ti-6Al-4V, Co-Cr-Mo, etc.) are used for medical applications. The addition of non-toxic elements such as Mo, Cu, Si, Zr and Mn also provides benefits, such as reducing the modulus of elasticity, increasing corrosion resistance and improving biocompatibility. In the present study, when choosing Ti-9Mo alloy, aluminum and copper (Cu) elements were added to it. These two alloys were chosen because one element is considered a favorable element for the body (copper) and the other element is harmful to the body (aluminum). By adding the copper alloy element to the Ti-9Mo alloy, the elastic modulus decreases to a minimum value of 97 GPa, and the aluminum alloy element increases the elastic modulus up to 118 GPa. Due to their similar properties, Ti-Mo-Cu alloys are found to be a good optional alloy to use.

## 1. Introduction

Biocompatible alloys are widely used in various biomedical applications due to their excellent mechanical properties and biocompatibility after implantation. Among different biocompatible alloys, titanium-based alloys have attracted significant attention in various uses of biocompatible parts due to their high strength-to-density ratio, corrosion resistance, and biocompatibility after implantation [1,2,3]. Among various titanium-based alloys, Ti-6Al-4V alloy is considered one of the most common biocompatible alloys due to its lower elastic modulus than stainless steel and cobalt–chromium alloys [4,5,6]. However, the presence of toxic elements such as aluminum and vanadium in the human body can lead to various health issues over time [7,8,9,10,11]. 

Reduced elastic modulus is a critical mechanical property for metallic biomaterials used in orthopedic applications, as it enables the implant to better match the mechanical properties of the surrounding bone tissue. When an implant has a higher elastic modulus than the surrounding bone tissue, it can result in stress shielding, which occurs when the implant bears the majority of the load instead of the bone [12]. This can lead to bone resorption and implant loosening, reducing the lifespan of the implant. Therefore, developing biocompatible Ti-Mo alloys with a reduced elastic modulus is a critical goal in the field of orthopedic biomaterials.

In addition, the use of software simulation has become increasingly popular in the field of materials science, as it provides a cost-effective and efficient way of predicting the behavior of materials before they are manufactured. In particular, the Finite Element Method (FEM) has been widely used to simulate the mechanical properties of materials and structures, including biomaterials [13,14]. FEM is a numerical method for solving partial differential equations that describe the behavior of a physical system. It subdivides the system into smaller and simpler parts, called finite elements, which are connected by nodes. The behavior of each element is described by a set of equations, and the solution of the entire system is obtained by combining the solutions of all elements. FEM has been successfully applied to simulate the behavior of various biomaterials, including dental implants, hip prostheses, and spinal implants [15,16,17]. To overcome the toxicity issues associated with conventional biocompatible alloys, various alloys have been developed in recent years. For example, titanium–manganese or titanium–molybdenum alloy groups have been developed by adding beta-phase stabilizers such as manganese and molybdenum to titanium alloys [18,19].

The increase in the beta phase in the presence of these elements leads to a decrease in the elastic modulus up to 100 GPa, making these alloys suitable for biomedical applications [20,21]. Furthermore, studies have been conducted to develop titanium, molybdenum, and copper alloys that can be used as medical alloys [22,23]. Development of new titanium alloys: Researchers are working on developing new titanium alloys with improved biocompatibility and mechanical properties. One such alloy is Ti-29Nb-13Ta-4.6Zr, which has shown promise in orthopedic applications [24,25]. Use of additive manufacturing: Additive manufacturing techniques, including 3D printing, are being used to create complex shapes and designs for biocompatible implants. This technology allows for precise control over the material’s microstructure and mechanical properties, leading to better performance and compatibility with the human body [26]. Advances in surface modification techniques: Surface modification techniques such as plasma spraying and electrochemical deposition are being used to improve the biocompatibility of existing alloys. These techniques can create a thin coating on the surface of the implant, which can enhance its osseointegration and corrosion resistance [27,28,29]. Exploration of new alloy elements: Researchers are investigating the use of new alloy elements such as zirconium, tantalum, and niobium in biocompatible alloys. These elements have shown potential in improving the mechanical and biological properties of the alloys [30,31]. Computational modeling and simulation: Advances in computational modeling and simulation are enabling researchers to predict the behavior of biocompatible alloys under different conditions. This technology can help optimize the design of biocompatible implants and improve their performance [32,33,34,35,36]. The effects of adding vanadium and nitrogen to Ti-15Mo alloy for biomedical applications were investigated by Kirmanidou et al. [37]. The results showed that the addition of vanadium and nitrogen led to the formation of a new beta phase in the microstructure, resulting in improved mechanical properties such as hardness and compressive strength. Li et al. explored the microstructure and mechanical properties of Ti-Mo-Nb alloys for biomedical applications [38]. Copper is known to have antimicrobial properties, and it has been used in various biomedical applications, such as dental implants and cardiovascular stents, with promising results. However, copper has also been shown to have potential biocompatibility concerns when used in the composition of metallic biomaterials. The intended duration of the implantation, the specific application, and the amount and form of copper can all affect the safety of using copper in the composition of metallic biomaterials [39].

In this study, Ti-9Mo alloy is selected as a base alloy, and different percentages of aluminum and copper are added to this composition using software simulation. The copper alloy element is considered in the composition because copper has favorable properties for the human body. Moreover, to investigate the effect of aluminum on the elastic modulus, this element is also considered. The main objective of this study is to investigate the behavior and effect of adding aluminum and copper to biocompatible Ti-Mo alloy using software simulation.

## 2. Materials and Methods

The present investigation involved the addition of copper and aluminum elements to the alloy composition using JMatPro 7.0 developed by the British Thermotech company, Louth, UK, a software application for engineering materials analysis and design. The choice of copper was based on its favorable biocompatibility [40], while titanium was chosen due to its known biocompatible properties. On the other hand, aluminum was not used as it is associated with various biological problems. The software environment enabled the definition of the compounds listed in Table 1 and extraction of the elastic modulus, density, strength, and hardness of the designed alloys in the form of comparative charts.

The simulation of density and elastic modulus was carried out with a Fraction Liquid coefficient of 0.01% and at temperatures ranging from 0 °C to 1800 °C. Mechanical properties analysis was performed after subjecting all samples to heat treatment at 720 °C and considering a variable grain size range of 1 µm to 20 µm.

## 3. Results and Discussion

### 3.1. Elastic Modulus

**Figure 1:**
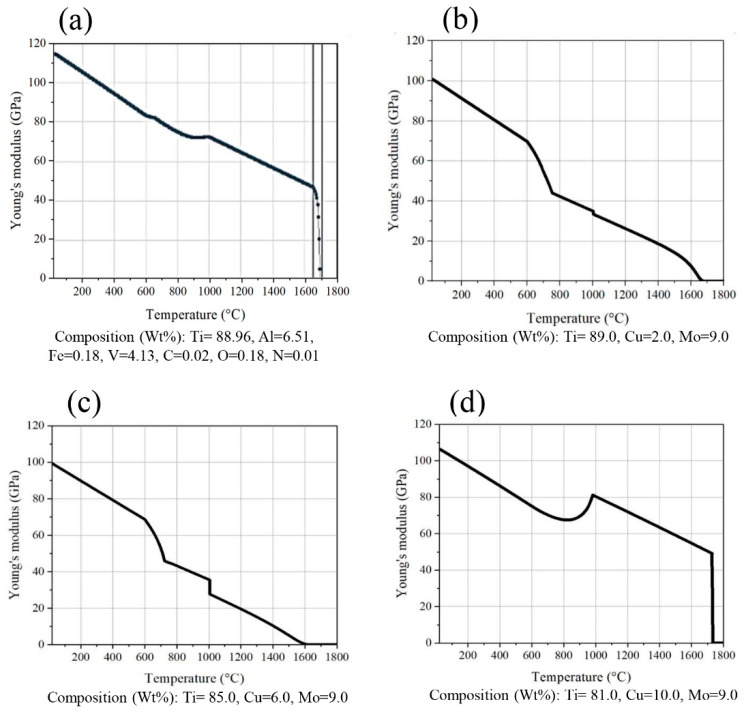
Comparison of different alloys including (**a**) Ti-6Al-4V, (**b**) Ti-2Cu-9Mo, (**c**) Ti-6Cu-9Mo and (**d**) Ti-10Cu-9Mo.

In Figure 1, the changes in elastic modulus with increasing temperature and rising copper (Cu) percentage are illustrated. The graph clearly shows that as the weight percentage of copper in the chemical composition of Ti-9Mo alloy increases, the value of the elastic modulus decreases. However, even with this decrease, the elastic modulus of Ti-10Cu-9Mo alloy (97 GPa) is still much higher than the model provided by Shuanglei for human bone (15–25 GPa) [41]. Additionally, Ti-10Cu-9Mo alloy has better elastic modulus conditions than the Ti-6Al-4V alloy (117 GPa). The reason why the elastic modulus decreases as the weight percentage of copper increases in the Ti-9Mo alloy is that copper atoms are larger than both titanium and molybdenum atoms. This size difference causes distortions in the crystal lattice of the alloy, making it easier for the metal to deform under stress, and resulting in a lower elastic modulus. In contrast, the Ti-6Al-4V alloy has a higher elastic modulus because it contains a higher percentage of the metal vanadium, which is a relatively small atom that does not cause significant distortions in the crystal lattice of the alloy. Therefore, even though Ti-10Cu-9Mo alloy has a lower elastic modulus than Ti-6Al-4V alloy, it still has better elastic modulus conditions for certain applications where a lower elastic modulus is desired, such as for bone implants [42].

**Figure 2:**
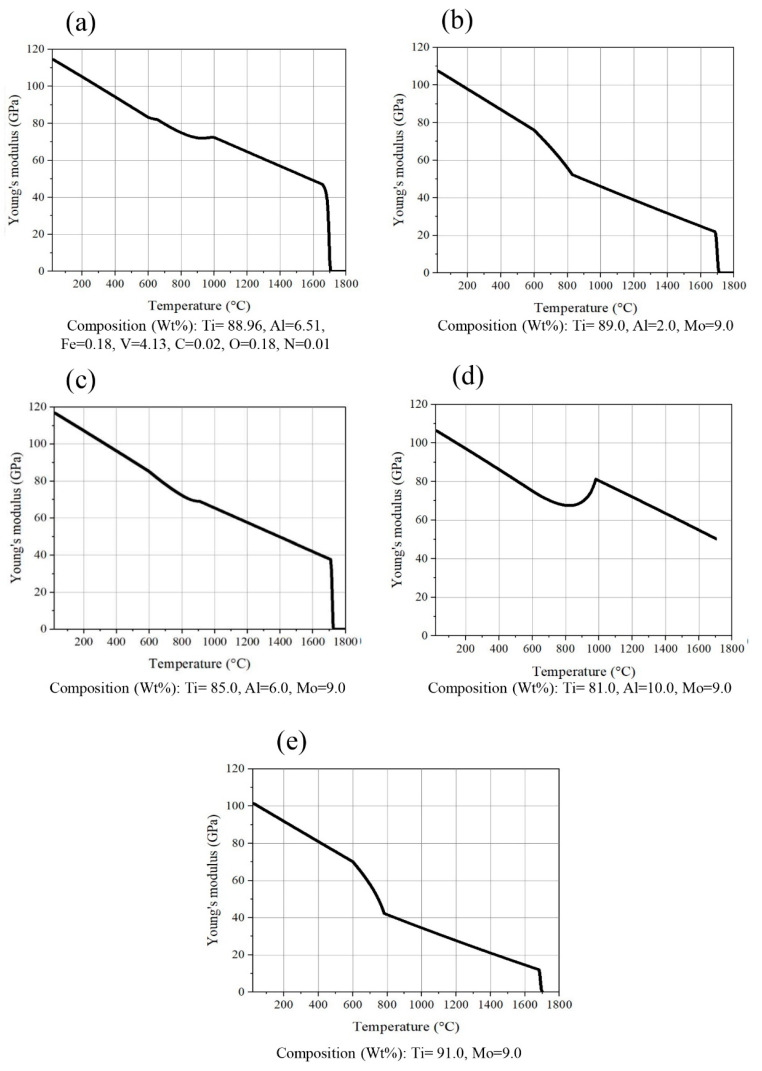
Comparative elastic modulus results for alloys (**a**) Ti-6Al-4V, (**b**) Ti-2Cu-9Mo-, (**c**) Ti-6Al-9Mo, (**d**) Ti-10Al-9Mo and (**e**) Ti-9Mo.

The present study investigates the comparative results of Ti-6Al-4V alloy with Ti-xAl-9Mo alloys, as depicted in Figure 2. The inclusion of aluminum in the chemical composition of Ti-9Mo alloy at various weight percentages has been observed to significantly enhance the elastic modulus. Notably, the elastic modulus increases up to 118 GPa, which is nearly equivalent to that of Ti-6Al-4V alloy. However, it should be noted that the detrimental effects of aluminum on Alzheimer’s [43] and the adverse effects of a high elastic modulus persist, despite the elimination of the negative impact of vanadium in Ti-xAl-9Mo alloys.

### 3.2. Comparison of Density Changes

**Figure 3:**
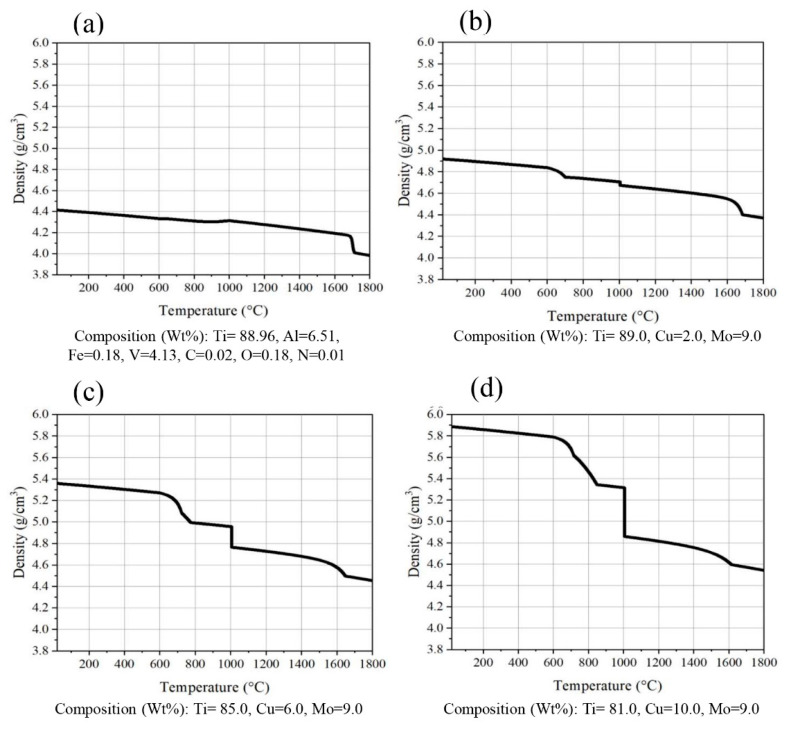
Density changes in alloys (**a**) Ti-6Al-4V, (**b**) Ti-2Cu-9Mo, (**c**) Ti-6Cu-9Mo and (**d**) Ti-9Mo-10Cu.

**Figure 4:**
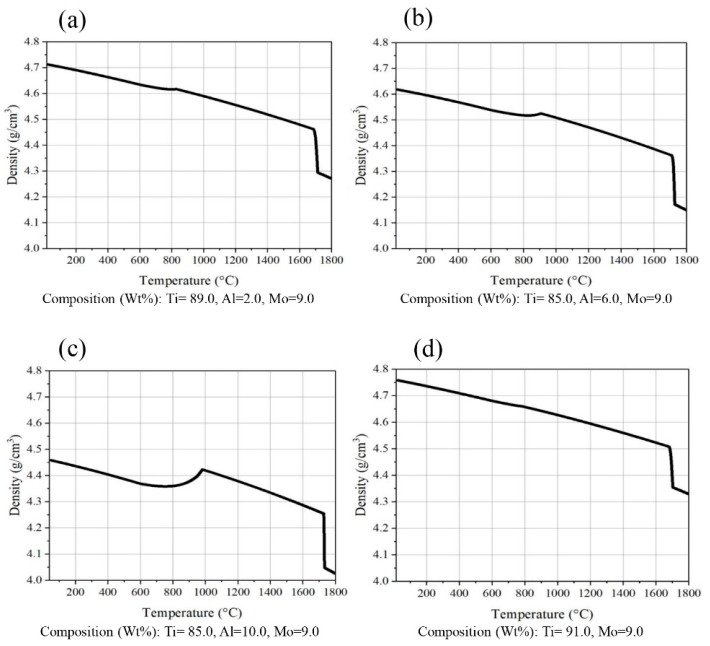
Density changes in alloys (**a**) Ti-2Al-9Mo, (**b**) Ti-6Al-9Mo, (**c**) Ti-10Al-9Mo, (**d**) Ti-9Mo.

The findings presented in Figure 3 indicate a comparison of various states of Ti-xCu-9Mo alloy with Ti-6Al-4V alloy. In general, the density of the alloy increases with the rise in the weight percentage of copper. For instance, with the addition of 10 weight percent of copper to the chemical composition, the density of the alloy is approximately 6 g per cubic centimeter. However, the increased density of the alloy may not be appropriate for certain medical purposes, such as hip joints, as the weight of the piece increases and may lead to patient discomfort. Furthermore, Figure 4 demonstrates a comparison of different states of Ti-xAl-9Mo-alloy with Ti-9Mo alloy. The density of the alloy did not significantly change with the addition of various weight percentages of aluminum. Notably, the density of all alloys containing aluminum is lower than that of alloys containing copper (Cu).

### 3.3. Changes in Hardness and Strength

**Figure 5:**
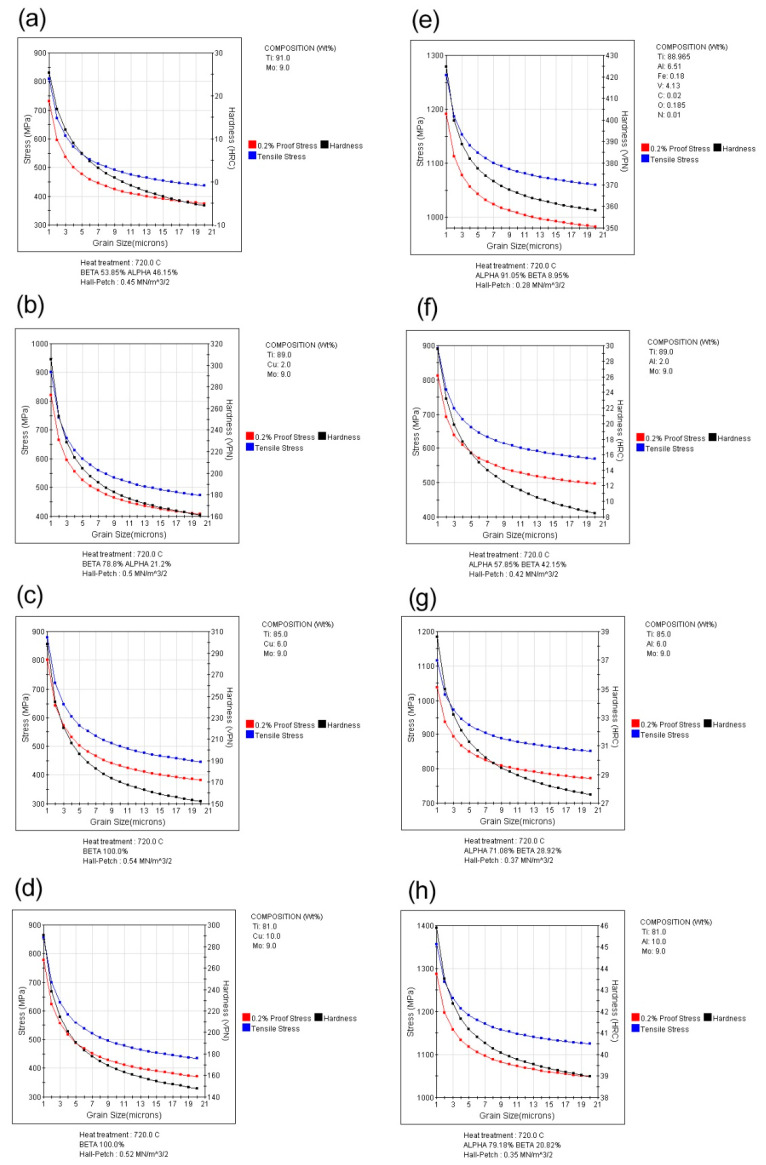
Comparison of hardness changes in different samples (**a**) Ti-9Mo, (**b**) Ti-2Cu-9Mo, (**c**) Ti-6Cu-9Mo and (**d**) Ti-10Cu-9Mo, (**e**) Ti-6Al-4V, (**f**) Ti-2Al-9Mo, (**g**) Ti-6Al-9Mo, (**h**) Ti-10Al-9Mo.

In Figure 5, a comparison of hardness, yield strength, and tensile strength of all examined samples concerning grain size changes is presented. The Ti-9Mo sample had a reported maximum tensile strength of 830 MPa, which increased with the addition of both copper and aluminum alloy elements. The analytical calculations of the software showed that the maximum available strength after adding 2% by weight of copper was recorded as 1220 MPa. Similarly, the maximum available strength after adding 10% by weight of aluminum was recorded as 1310 MPa.

**Figure 6:**
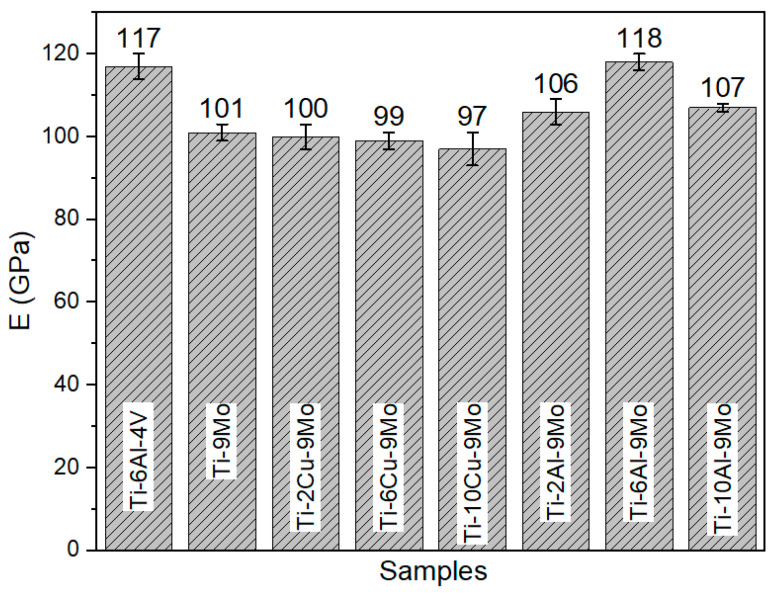
Comparison of elastic modulus results from software analysis.

Figure 6 illustrates a summary comparison of elastic modulus values. As depicted in the figure, the lowest elastic modulus among the investigated alloys belongs to Ti-10Cu-9Mo alloy, with a value of 97 GPa, which is approximately 20 GPa less than that of Ti-6Al-4V alloy. This reduction in the elastic modulus can potentially alleviate the patient’s pain in the long term [44]. In metal alloys, Young’s modulus is directly related to the equilibrium interatomic distance and the balance interatomic distance to the lattice parameter. The addition of certain metal elements, such as manganese, can decrease the inter-atomic distance by reducing the lattice parameter. Consequently, Young’s modulus increases with the decrease in the inter-atomic distance [45]. In this study, an increase in aluminum content led to a significant rise in the elastic modulus, whereas copper caused a decrease in the elastic modulus [46]. However, such a decrease was not observed in the current research.

**Figure 7:**
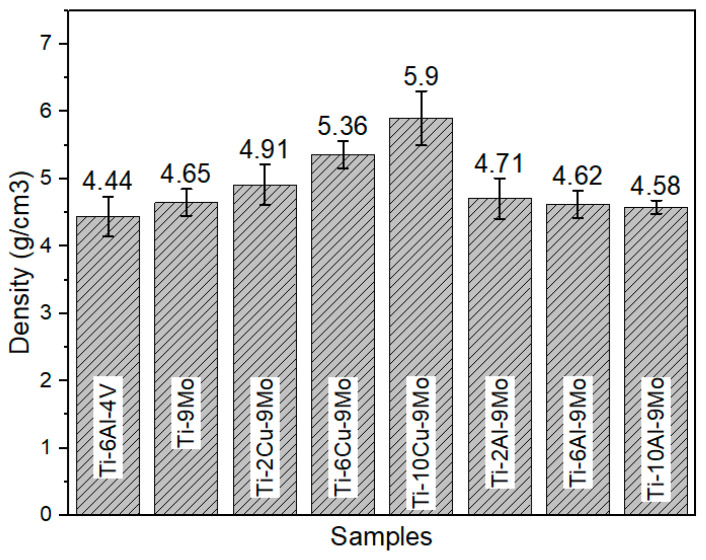
Comparison of density changes in the examined alloys results.

The data presented in Figure 7 provide a comparison of the density values of the investigated alloys at ambient temperature (25 °C). As evident from the diagram, the density of the alloy increases with the increase in the weight percentage of copper (Cu), and adding up to ten weight percent of copper increases the density of the alloy to approximately 6 g per cubic centimeter. This value is 1.6 g per cubic centimeter more than that of the Ti-6Al-4V alloy. It is worth noting that according to Equation (1), which relates density to mass and volume, the mass number of copper (Cu) is approximately 2.3 times that of aluminum. Therefore, the density of the alloy increases significantly after the addition of copper (Cu).

(1) $$\rho =\frac{n\cdot M}{V_{unit.cell}\;N_{a}}$$

In this equation, *n* is the number of atoms belonging (attached (to the unit cell, *V* is the volume of the unit cell, *M* is the atomic weight of the metal, and *N_a_* is Avogadro’s number.

**Figure 8:**
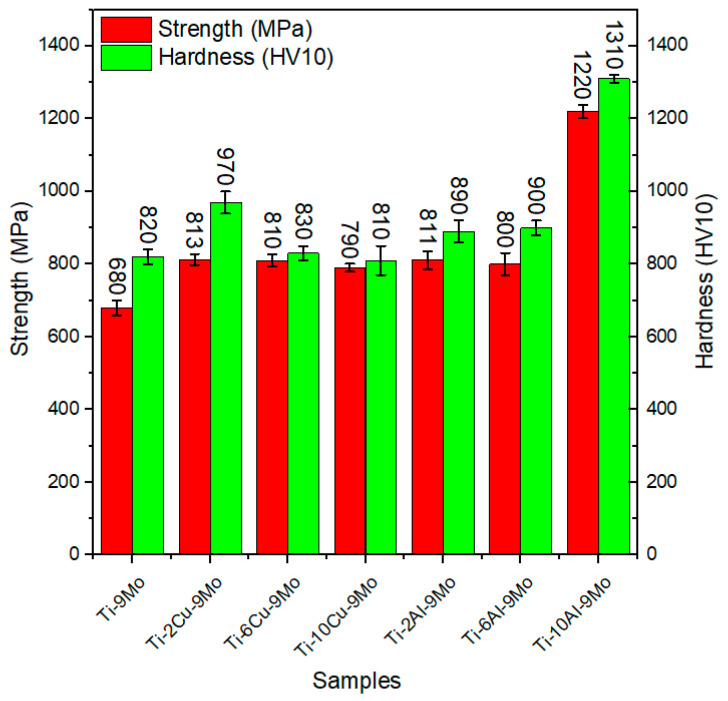
Changes in the strength and hardness of the alloy in the software environment.

**Figure 9:**
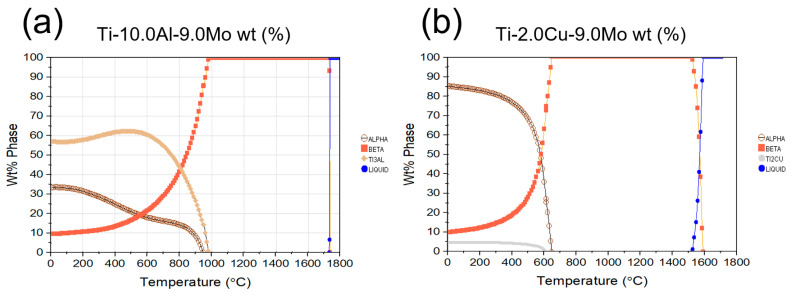
Phases formed in the alloy with increasing temperature (**a**) Ti-10Al-9Mo- alloy and (**b**) Ti-2Cu-9Mo alloy.

Figure 8 presents a comparison of the tensile strength and hardness results at ambient temperature among the examined alloys. The simulation results revealed the presence of intermetallic compounds in the alloys, which can be considered as one of the reasons for the increase in strength. By increasing the weight percentage of aluminum to ten weight percent, the presence of Ti-3Al-9Mo compound was observed in the software analysis, which can resist crack movement and increase strength. This observation was also confirmed in copper (Cu) alloys. The percentage of phases formed at ambient temperature is a significant factor in determining the difference in strength. Figure 9a illustrates that the presence of Ti-3Al-9Mo intermetallic compound constitutes approximately 60% of the alloy, leading to an increase in strength, as well as resistance to crack growth, propagation, and dislocation movement. Conversely, Figure 9b reports that the amount of intermetallic compound Ti-3Cu-Mo is less than ten percent by weight, resulting in a disparity in strength between the two alloy compounds. In Figure 9b, the presence of Ti-2Cu-9Mo compound was established, which is also a strengthening factor. Previous studies have mentioned the Ti-2Cu-9Mo phase, which can enhance the antibacterial ability of the alloy [47,48,49,50].

## 4. Conclusions

In this study, a simulation-based investigation of the influence of aluminum and copper on the mechanical properties of biocompatible Ti-Mo alloys. The results showed that the addition of aluminum and copper can significantly improve the strength and hardness of Ti-Mo alloys while reducing their elastic modulus. The findings of our study have important implications for the development and production of metallic biomaterials for orthopedic and biomedical applications. By understanding the effects of different compositions on the mechanical properties of biocompatible Ti-Mo alloys, implants and other biomedical devices can improved in terms of design and performance. However, it is important to note that this study has limitations and there are areas that require future research. This simulation was conducted under specific testing conditions, and experimental validation is needed to confirm our results. Additionally, further investigation is needed to explore the effects of different compositions, processing parameters, and testing conditions on the mechanical properties of biocompatible Ti-Mo alloys. The present study utilized a software environment for alloy design and simulation to improve the properties of Ti-xMo alloys, which are commonly used for biocompatible parts but suffer from low strength and hardness. The results can be concluded as follows:

1. The addition of copper to Ti-9Mo alloy leads to a decrease in elastic modulus with a minimum value of 97 GPa, while the addition of aluminum increases the modulus to 118 GPa.

2. The inclusion of both copper and aluminum alloy elements to Ti-9Mo alloy enhances the strength and hardness in all states, with a significant increase in strength observed in aluminum alloys due to formed phases.

3. Alloys with copper have higher density than those with aluminum due to the atomic characteristics of these elements. Copper has a larger atomic mass, making each copper atom heavier. When added to an alloy, this increases the total density. Additionally, copper atoms are more densely packed than aluminum in a crystal lattice structure, further contributing to the higher density of copper-containing alloys.

4. Due to the risk of Alzheimer’s disease associated with aluminum alloys and the increase in elastic modulus observed in all weight percentages examined, copper-containing alloy compositions are considered useful due to their favorable strength and hardness parameters, along with a decreased elastic modulus by 20 GPa compared to Ti-6Al-4V alloy. Additionally, copper is an essential element for the human body with no human health risks.

5. Ti-2Cu-9Mo and Ti-6Cu-9Mo alloys exhibit the best elastic modulus, strength, and hardness among the examined alloys.

## Figures and Tables

**Table 1:** Chemical compounds analyzed in the software environment.

Composition (wt.)	Ti	Mo	Cu	Al	V
Ti-2Cu-9Mo	Base	9	2	0	0
Ti-6Cu-9Mo	Base	9	6	0	0
Ti-10Cu-9Mo	Base	9	10	0	0
Ti-2Al-9Mo	Base	9	0	2	0
Ti-6Al-9Mo	Base	9	0	6	0
Ti-10Al-9Mo	Base	9	0	10	0
Ti-6Al-4V	Base	0	0	6	4
